# Beige Fat Maintenance; Toward a Sustained Metabolic Health

**DOI:** 10.3389/fendo.2020.00634

**Published:** 2020-09-04

**Authors:** Atefeh Rabiee

**Affiliations:** Department of Chemical and Systems Biology, Stanford University, Stanford, CA, United States

**Keywords:** brown fat, beige fat, development and origin, transcriptional regulation, epigenetic regulation, browning, thermogenesis, maintenance

## Abstract

Understanding the mammalian energy balance can pave the way for future therapeutics that enhance energy expenditure as an anti-obesity and anti-diabetic strategy. Several studies showed that brown adipose tissue activity increases daily energy expenditure. However, the size and activity of brown adipose tissue is reduced in individuals with obesity and type two diabetes. Humans have an abundance of functionally similar beige adipocytes that have the potential to contribute to increased energy expenditure. This makes beige adipocytes a promising target for metabolic disease therapies. While brown adipocytes tend to be stable, beige adipocytes have a high level of plasticity that allows for the rapid and dynamic induction of thermogenesis by external stimuli such as low environmental temperatures. This means that after browning stimuli have been withdrawn beige adipocytes quickly transition back to their white adipose state. The detailed molecular mechanisms regulating beige adipocytes development, function, and reversibility are not fully understood. The goal of this review is to give a comprehensive overview of beige fat development and origins, along with the transcriptional and epigenetic programs that lead to beige fat formation, and subsequent thermogenesis in humans. An improved understanding of the molecular pathways of beige adipocyte plasticity will enable us to selectively manipulate beige cells to induce and maintain their thermogenic output thus improving the whole-body energy homeostasis.

## Introduction

Prolonged periods of excess energy storage lead to weight gain and obesity. This excess energy is stored in white adipose tissue (WAT), which is the major fat storage depot and is linked to metabolic disease states. Contrarily, brown adipose tissues (BAT) dissipates energy as heat and consequently modulates daily energy expenditure (1). As the amount of metabolically active BAT is limited in patients with obesity and type two diabetes (T2D), alternatives are required to increase energetic expenditure via thermogenesis (2–6). In addition to classic brown adipocytes, human adults have inducible brown adipocytes (named as brown-in-white or beige) with unique characteristics that differentiate them from both white and brown adipocytes (1, 7–10). Inducing beige adipocytes formation in WAT (browning) potentially decreases the negative effects of excess WAT and improves overall metabolic health (11). In response to cold exposure, inducible BAT greatly increases mitochondria and uncoupling protein 1 (UCP1) abundance. Additionally, after browning stimuli are removed, there is a rapid decrease of the thermogenic gene expression. This dynamic response in beige fat can be contrasted with classic BAT where the levels of UCP1 and mitochondria are constitutively high (12, 13). Although numerous studies have identified several regulators of browning and thermogenesis, the molecular basis underlying beige adipocyte reversibility is yet to be understood (14–20).

## Development and Origin of Brown and Beige Adipocytes

Beige adipocytes mainly reside in subcutaneous white adipose tissue (scWAT) depots (8). The scWAT depots in humans include cranial, facial, abdominal, femoral, and gluteal depots. In rodents, scWAT includes the anterior subcutaneous white adipose tissues (ascWAT) and the posterior subcutaneous adipose tissue (pscWAT) which itself includes inguinal, gluteal, and dorso-lumbal WAT (7, 21). BAT depots are distributed in the thoracic (mediastinal) and scapulae (interscapular, cervical, and axillary) areas of mice and rats (22). In humans, BAT was initially thought to exist only in the neck and shoulder of infants (23). However, later studies found active BAT in the paracervical and supraclavicular as well as in the anterior neck regions of adult humans (23–27).

In mammals, BAT is formed earlier during embryogenesis as compared to WAT. In human fetuses BAT formation begins early in the second trimester primarily in the head and neck regions and later in development forms in the trunk as well as in upper and lower limbs. The development of subcutaneous white adipose tissue is completed prenatally (28). In rodents, functional thermogenic BAT is formed 2 days before birth (E18–19) (29–32) and the development of scWAT continues postnatally (33–35).

Both white and brown adipose tissues are known to have mesodermal origins including the intermediate and lateral plate as well as the axial, and paraxial mesoderm. The paraxial mesoderm gives rise to BAT (36), and though the origin of scWAT is still debated, the progenitors of scWAT are known to originate from both the mesoderm and neuroectoderm (37–40). Furthermore, each fat depot includes numerous distinct progenitor fields that vary with age, gender, and environmental conditions. Additionally, scWAT depots are mainly derived from paired related homeobox 1 (PRX1) expressing progenitors (41–45). Despite the previous view that myogenic factor 5 (MYF5), paired box 7 (PAX7), and paired box 3 (PAX3) expressing progenitors only give rise to BAT, it is now believed that the scWAT depots of the dorsal–anterior body region originated partly from those progenitors (9, 39, 46–49). While *ex vivo* studies reported the presence of both PDGFRα and PDGFRβ in adipocyte stem cells (ASCc) (50), in adult mouse progenitors are heterogeneous and either express PDGFRα or PDGFRβ (51, 52). A recent study by Gao et al. suggested that the balance between PDGFRα and PDGFRβ determines whether progenitors will commit to beige (PDGFRα) or white (PDGFRβ) adipocytes (53). Some satellite cell-derived myoblasts in skeletal muscle and fibro-adipogenic progenitors (FAPs) also give rise to beige fat with higher rate of glycolysis and hence, named as glycolytic beige adipocyte (54).

## Transcriptional Regulation of Brown and Beige Adipocytes

In brown and beige adipocytes, the adipogenic differentiation program and the thermogenic program are regulated by different pathways each involving a complex network of transcription factors (TFs) and epigenetic factors. The main parts of transcriptional machinery regulating fat cell differentiation is common among various types of fat cells and has been extensively discussed elsewhere (8, 55–57). Here, several TFs which are the main signatures of brown and beige fat are briefly described.

### EBF2

Early beta Cell factor 2 is a marker of committed brown adipocytes in rodents and humans (29, 57, 58). EBF2 promotes brown adipocytes differentiation by recruiting peroxisome proliferator-activated receptor gamma (PPARγ) to its brown-specific chromatin regions (58). Overexpression of EBF2 in myoblast induces brown adipogenesis and inhibits myogenesis (30). In the absence of EBF2, the brown fat specific features of BAT are abolished (58). Moreover, overexpression of EBF2 in WAT induces browning and thermogenesis (59).

### PRDM16

PR domain zinc finger 16 promotes brown and beige adipocyte differentiation and inhibits myogenic and WAT gene expression in mice and *in vitro* in human fibroblasts (46, 60–63). PRDM16 is also important in brown fat maintenance by binding to specific enhancer regions along with a mediator complex to establish enhancer-promoter looping, leading to the expression of PPARα and PPARγ co-activator 1A (PGC1α). Also, PRDM16 interacts with PGC1α (described below) and increases its transcription (60, 64, 65). PRDM16 also inhibits the signaling of repressor type 1 interferon response genes thereby preventing mitochondrial dysfunction and a decrease in UCP1 levels (66). PRDM16 overexpression increases beige adipogenesis and thermogenesis in WAT and its deficiency inhibits beige adipocyte formation (67, 68).

### PGC1α

PPARγ co-activator 1A is known to directly interact with PRDM16 and PPARγ in brown adipocytes (60, 69). In differentiated brown and beige adipocytes, PGC1α plays a crucial role in cold-induced thermogenesis and thermogenic maintenance in mice and human WAT (69, 70). Interacting with other transcriptional regulators, PGC1α activates the transcription of UCP1 and several mitochondrial genes (65, 71, 72). PGC1α overexpression induces thermogenesis in adipocytes and myocytes (73, 74). Brown adipocytes lacking PGC1α express lower levels of UCP1 in response to adrenergic stimuli (75, 76). PGC1α is also required for the browning of WAT (77).

### IRF4

Interferon regulatory factor 4 interacts with PGC1α upon cold stimuli and regulates UCP1 expression by binding to its chromatin regulatory regions in BAT of mice and humans (78).

### ZFP516

Zinc finger protein 516 also increases brown adipogenesis and thermogenesis upon cold induction. ZFP516 interacts with PRDM16 and activates UCP1 and PGC1α expression (79).

### CREB-ATF2

Phosphorylation and activation of activating transcription factor 2 (ATF2) and cAMP-responsive element-binding (CREB) downstream of cold-induced adrenergic signaling results in activation of UCP1 and PGC1α gene expression (80).

### KLF11

Kruppel-like factor 11 expression is induced *in vitro* in human white adipocytes in response to the PPARγ agonist rosiglitazone and promotes beige adipocyte-selective gene expression via increasing PPARγ binding to beige-selective super-enhancers (81).

### FOXC2

Forkhead box protein C2 expression increases beige adipocyte formation by promoting protein kinase A (PKA) activity; a main kinase activated downstream of the adrenergic pathway upon cold induction (82).

### FOXP1

Foxhead P1 expression is highly enriched in vWAT and acts as a transcriptional repressor that directly represses β3-AR transcription. FOXP1 deletion increases brown fat activation and the browning of WAT and its overexpression is inhibitory to browning and thermogenesis in mice and humans (83).

### GABPα

GA-binding protein α is expressed in myoblasts and inhibits myogenesis and promotes adipogenesis and beige fat development. The interaction between PGC1α and GABPα is also shown to stimulate mitochondria biogenesis and oxidative phosphorylation (84–86). GABPα expressing beige adipocytes are unlike other beige adipocytes with higher glucose oxidation rate than fatty acid oxidation (54).

EBF2 and GABPα mainly function as commitment factors. EBF2 marks committed brown adipocytes and GABPα inhibits myogenic development thereby promoting brown/beige fat formation. During brown fat differentiation, EBF2, PRDM16, and ZFP516 together with other common adipogenic regulators such as C/EBPβ and PPARγ regulate the induction of brown fat specific genes with PRDM16 acting mainly as a coactivator. Upon cold exposure and activation of adrenergic signaling, the concentration of cyclic AMP (cAMP) and PKA activity increase. PKA activity, which is further increased by FOXC2, results in p38 MAPK phosphorylation and activation. Phosphorylated p38 then phosphorylates and activates both ATF2 and PGC1α. IRF4 recruits phosphorylated PGC1α to the chromatin which will then coactivate PPARγ, thyroid receptor (TR), and retinoid X receptor alpha (RXRα) to increase the expression of thermogenic genes.

Several other transcriptional regulators and nuclear receptors are known to be involved in activating or repressing brown and beige fat specific programs (87). The roles of long non-coding RNA and microRNA in brown/beige fat regulation are comprehensively discussed elsewhere (88, 89).

## Epigenetic Regulation of Brown and Beige Adipocytes

The chromatin landscape which plays a critical role in brown/ beige fat identity, differentiation, and activation is modulated via tight cooperation between TFs and epigenetic modifiers. ChIP-seq analysis showed that PPARγ bind to 55% similar regions in BAT, scWAT, and vWAT. However, only 10% of PPARγ binding sites are BAT specific (90). In a separate study in human adipocytes, PPARγ ChIP-seq before and after rosiglitazone induced browning identified only 10% of the binding sites were different, indicating that the beige and BAT-selective characteristics are derived from a small subset of genomic sites (81, 90). Nuclear isolation and chromatin analysis of specific cell types from heterogeneous adipose tissue using UCP1-NuTRAP mice demonstrated the stability of the chromatin landscape in BAT upon temperature changes while extreme plasticity of chromatin landscape was observed in beige adipocytes with temperature changes (91).

Enhancers in BAT, but not WAT are enriched in active histone marks such as H3K4me1/2 and H3K27ac (92). The UCP1 promoter in BAT is enriched for H3K4me3 and in WAT is enriched for H3K27me3 (93). Several histone-modifying enzymes have been identified that regulate the chromatin landscape in brown fat (94, 95). Some examples are ubiquitously transcribed tetratricopeptide repeat X chromosome (UTX), lysine-specific histone demethylase 1 (LSD1), jumonji domain containing 3 (JMJD3), and jumonji domain containing 1A/lysine demethylase 3A (JMJD1A/KDM3A) demethylate H3K27me3 at the promoters of UCP1 which leads to the upregulation of BAT selective genes in response to acute cold exposure (93, 96–98). Deletion of myeloid/lymphoid or mixed-lineage leukemia 4/lysine demethylase 2D (MLL4/KMT2D) in brown precursors increases the level of repressor H3K27me3 marks and impairs brown adipogenesis in mice and humans (99). Ablation of euchromatic histone-lysine N-methyltransferase 1 (EHMT1) decreases activator H3K4me3 marks and impairs brown adipocyte differentiation thereby activating myogenesis (62). The depletion of histone deacetylase 3 (HDAC3) in mice decreases thermogenesis in cold temperatures (100). In general, cold induction in brown adipocytes increases the expression of activator H3K27ac marks and removes the repressive H3K27me3 marks (101).

## Browning: Paths and Players

**Figure 1:**
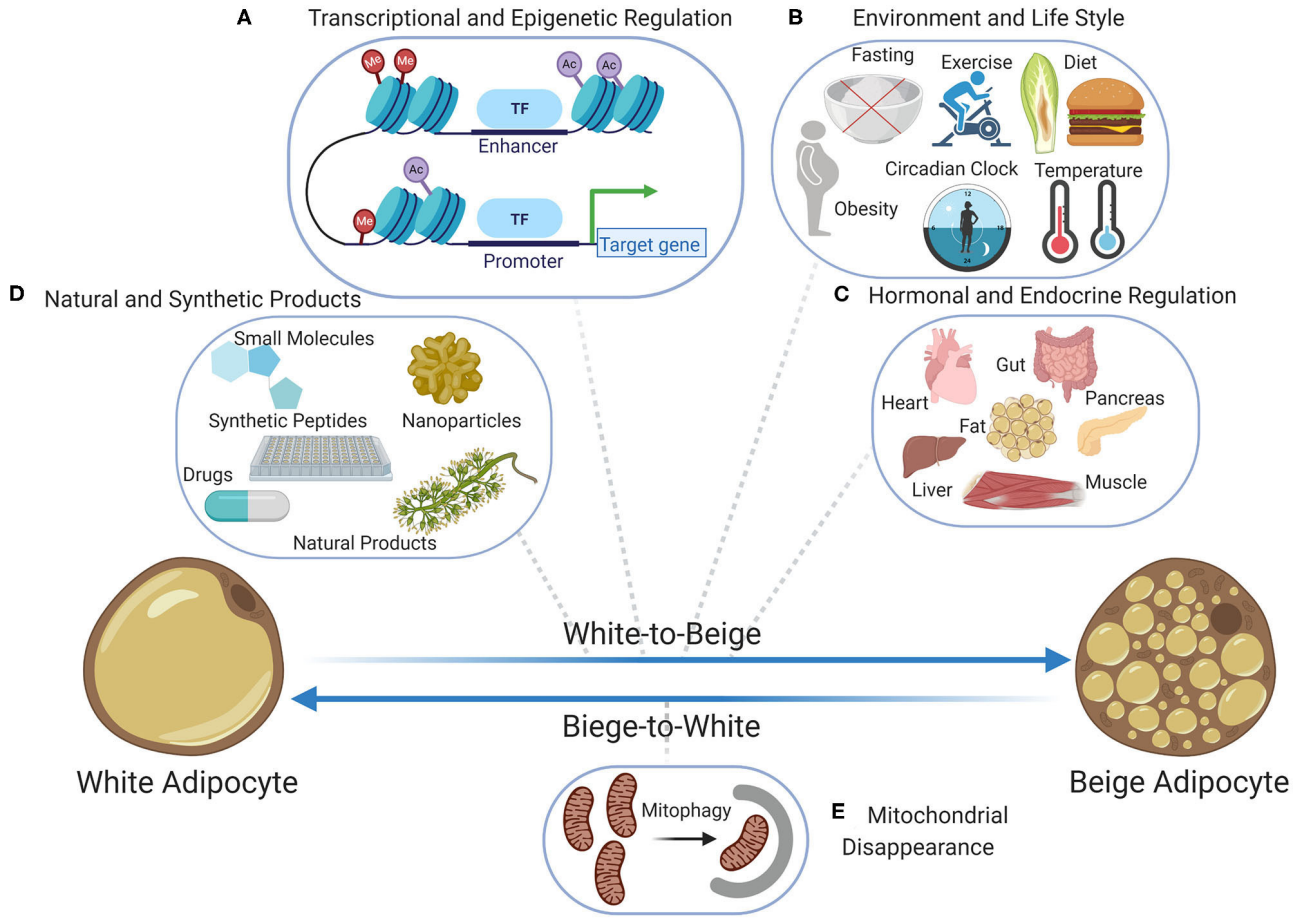
Bidirectional transition between beige and white adipocytes; the beige-to-white transition (browning) has been extensively studied at the levels of **(A)** transcriptional and epigenetic regulation including the chromatin landscape, transcriptional regulators, and epigenetic modifiers, **(B)** the role of lifestyle and environment including diet, fasting, obesity, exercise, temperature, and circadian rhythm, **(C)** the role of endocrine factors and hormones secreted by various organs including pancreas, muscle, liver, heart, gut, and fat when adapting to environmental challenges, **(D)** the role of natural products and plant extracts as well as the role of synthetic chemical products including small molecules, nanoparticles, synthetic peptides, and drug. Contrarily, the beige-to-white transition which is the immediate result of stimuli removal is poorly investigated and so far, **(E)** mitochondrial disappearance (mitophagy) is known to be the main contributor. Figure created with ©BioRender.io.

The increase of UCP1 positive, multilocular, thermogenic beige adipocytes within WAT (browning) is a potential therapeutic approach to increase insulin sensitivity and combat metabolic diseases such as obesity (102, 103). In mammals all browning features can be achieved by adrenergic stimulation, the main signaling pathway of thermogenic BAT which is induced by cold temperatures. In addition, several alternatives to this canonical pathway have been reported to regulate browning of WAT through interorgan crosstalk (104). Several browning agents have been reported that are extensively reviewed elsewhere (20, 105–107) (Figure 1). For example, numerous pharmacological small molecules, dietary compounds, and nutritional agents are known to increase WAT browning (108–111). Additionally, various organs respond to environmental challenges such as cold, fasting, feeding, and exercise by secreting several factors and hormones that contribute to the browning (3, 112). Gut microbiota as well as immune cells and macrophages influence WAT browning process and have been well-discussed by others (113–115). In mammals, increased energy expenditure and browning of WAT after gastric bypass surgery have been reported (116–118). The link between WAT browning and thermogenesis is supported by generic mouse models of UCP1 knockout and BAT paucity, both leading to compensatory browning of WAT (119, 120).

Beige adipocytes form via three main processes (121): (I) proliferation and *de novo* differentiation of beige adipocytes from the progenitor pool located in adipose vasculature mural cells and express smooth muscle actin (SMA), myosin-11 (MYH-11), and PDGFRα (122, 123). In addition to adipose tissue vasculature, smooth muscle cells are also proposed as a source of beige progenitors (124); (II) transdifferentiation of mature white adipocytes to beige adipocytes (12, 125) where adrenergic stimulation by cold and high-fat diet feeding increases *de novo* formation of beige adipocytes as well transdifferentiation of mature white cells into beige adipocytes (16, 51, 126, 127); (III) the activation of dormant beige adipocytes without the involvement of progenitors contributes to the formation of thermogenic beige fat (12, 128, 129). These competing hypotheses remain unresolved as current lineage tracing technologies are unable to distinguish between white-to-beige adipocyte transdifferentiation and the activation of dormant beige cells. The current strategies for lineage tracing are summarized by Sebo and Rodeheffer (129).

## WAT Browning in Humans

Recent studies that carried out FDG-PET/CT imaging were able to identify BAT activity in supraclavicular, cervical, axillary, and less often in abdominal, mediastinal, and paraspinal fat depots. The UCP1 positive fat depots in adult humans detected as ^18^F-FDG positive contained both classical brown adipocytes as well as beige adipocytes (6, 11, 24, 26, 130–135). Beige and brown fat activity in humans is increased in response to cold exposure and is inversely associated with age, body mass index, and the levels of circulating lipid and glucose (24, 25, 136–141). Studies have shown that even in lean people with larger BAT depot, the cold induced thermogenic function of BAT does not significantly impact energy balance (142, 143). Hence, targeting the large scWAT for browning to increase thermogenesis has recently become a target for therapeutic approaches. So far, the browning of WAT in humans has only been reported under extreme conditions and its contribution to energy expenditure compared to BAT is minor (144–146). Ten days of cold exposure in humans was insufficient to induce WAT browning, despite increasing BAT activity. This indicates a requirement for higher levels of adrenergic stimuli (147, 148). The effects of prolonged physical training on human scWAT browning and increasing the levels of circulating adiponectin, apelin, irisin, and FGF21 are in line with improved metabolic health (145, 149). Additionally, severe weight loss in cancer patients as well as in obese patients going through weight loss surgeries leads to increased browning (150, 151). Prolonged elevations in norepinephrine levels as a consequence of burn injury also leads to increased scWAT browning and thermogenesis (152). Surgical trauma, not necessarily related to the incision, is also linked to local and distal WAT browning in humans (153).

## Beige Reversibility and Maintenance

The thermogenic phenotype of beige adipocytes is reversible upon withdrawal of the external stimuli. Upon removal of adrenergic stimulation (for example in warm temperature), beige fat will gradually convert into cells with a unilocular lipid droplet and will progressively lose beige characteristics while increasing the white characteristics (e.g., reduced mitochondria and thermogenesis). This beige-to-white transformation is accompanied by reduced innervation, vasculature, and UCP1 expression and increased neural chemorepellent (semaphorin III) secretion and leptin expression (154–156). Although beige phenotype reversibility seems like a recent hallmark of adipocyte plasticity, it has been reported for decades (157–160). The phenotypic and morphological conversion found in beige fat upon withdrawal of stimuli is not observed in classical brown adipocytes (46). In 2013, Christian Wolfrum's research team used lineage tracing to validate beige-white interconversion. They showed that the cold-induced beige fat was reversed within 5 weeks of warm temperature and almost 75% of the whitened beige adipocyte could become beige again upon cold induction. Interestingly, after a second cold exposure, half of the beige adipocytes were formed from the former whitened beige adipocytes and the other half of the newly formed beige adipocytes seemed to come from a different source (12). Though the beige adipocytes lost their brown-like phenotype and acquired a white-like phenotype when the temperature was increased, they kept their epigenetic memory of the cold exposure which allowed them to activate browning genes as soon as they were exposed to cold temperatures (91). Interestingly, beige fat apoptosis and death was not found to be the cause of beige phenotype loss (12). Contrarily, BAT whitening was shown to increase cell death by increasing adipose inflammation, indicating a lack of plasticity in BAT (161). In 2015, Kozak and his research team reported much higher dynamics in UCP1 and mitochondrial turnover in beige fat when compared to BAT (162). In 2016, Kajimura and his research team elegantly linked the beige-to-white transition to mitochondrial disappearance (mitophagy). Mitophagy increased upon adrenergic stimuli withdrawal and was shown to be mediated by parkin (PARK2) recruitment to the mitochondria. Inhibition of autophagy via deletion of autophagy-related 5 (ATG5), autophagy-related 12 (ATG12), and PARK2 maintained the beige phenotype after stimuli removal. By monitoring single-cells, the same study also observed direct transdifferentiation from beige-to-WAT which did not involve an intermediate step (163). Recently, a natural and more stable beige adipose depot called thigh adipose tissue (tAT) was identified in mice (164). In contrast to classic beige adipocytes, tAT seems rather stable and maintains a beige fat phenotype in warm temperatures. However, high-fat diet (HFD) feeding and aging increased the white phenotypic features of tAT including the presence of unilocular adipocytes. Browning stimuli can increase brown adipocyte gene expression in tAT to a higher level than in iWAT. Furthermore, tAT has a higher rate of energy expenditure and lower expression of inflammatory genes relative to iWAT (164).

## Future Directions and Perspectives

With the worldwide increase in obesity and its comorbidities, many studies have indicated the great potential of beige fat to increase energy expenditure. The main bottleneck in the use of beige adipocytes as a therapeutic target is the fact that the beige adipocytes transition to a white phenotypic state shortly after stimuli withdrawal. Studies in mice have shown that obesity increases the beige-to-white adipocyte transition (165, 166). The instability of beige adipocyte compared to classic BAT could be partly explained by differences in their developmental origins. Chromatin and epigenetic analysis during cold to warm thermal shifts uncovered that the chromatin landscape is stable in BAT while in beige adipocytes chromatin changes are transient (91). Currently mitophagy is thought to be the main contributor to beige-to-white transition (Figure 1), and a higher rate of mitochondrial biogenesis or a lower rate of mitophagy could explain BAT stability when compared to beige adipocytes. Understanding the underlying mechanism of thermogenic maintenance in BAT and tAT after external stimuli removal may elucidate the pathways that prevent the beige-to-white transition. In this regard, strategies to inhibit mitochondrial autophagy as well as to increase mitochondrial biogenesis are potential therapeutic prospects to prolong the thermogenic phenotype of beige adipocytes. Beige adipocyte maintenance has the potential to attenuate reoccurring weight gain after weight loss surgery, diet, and exercise.

## Author Contributions

The author confirms being the sole contributor of this work and has approved it for publication.

## Conflict of Interest

The author declares that the research was conducted in the absence of any commercial or financial relationships that could be construed as a potential conflict of interest.
